# Author Correction: Comprehensive genomic characterization of HER2-low and HER2-0 breast cancer

**DOI:** 10.1038/s41467-023-44124-y

**Published:** 2023-12-14

**Authors:** Paolo Tarantino, Hersh Gupta, Melissa E. Hughes, Janet Files, Sarah Strauss, Gregory Kirkner, Anne-Marie Feeney, Yvonne Li, Ana C. Garrido-Castro, Romualdo Barroso-Sousa, Brittany L. Bychkovsky, Simona DiLascio, Lynette Sholl, Laura MacConaill, Neal Lindeman, Bruce E. Johnson, Matthew Meyerson, Rinath Jeselsohn, Xintao Qiu, Rong Li, Henry Long, Eric P. Winer, Deborah Dillon, Giuseppe Curigliano, Andrew D. Cherniack, Sara M. Tolaney, Nancy U. Lin

**Affiliations:** 1 Medical Oncology, Dana-Farber Cancer Institute, Boston, MA USA; 2 Breast Oncology Program, Dana-Farber Brigham Cancer Center, Boston, MA USA; 3 Harvard Medical School, Boston, MA USA; 4 Department of Oncology and Hematology-Oncology, University of Milano, Milano, Italy; 5 Broad Institute of Harvard and MIT, Cambridge, MA USA; 6 Dasa Institute for Education and Research (IEPD), Brasilia, Brazil; 7 Dasa Oncology/Hospital Brasilia, Brasilia, Brazil; 8 Division of Cancer Genetics and Prevention, Dana-Farber Cancer Institute, Boston, MA USA; 9 Yale Cancer Center, Yale School of Medicine, Smilow Cancer Hospital, New Haven, CT USA; 10 Department of Pathology, Brigham and Women’s Hospital, Boston, MA USA; 11 Division of Early Drug Development, European Institute of Oncology IRCCS, Milano, Italy; 12 Present Address: Department of Pathology, Weill Cornell Medicine, New York, NY USA

**Keywords:** Breast cancer, Breast cancer

Correction to: *Nature Communications* 10.1038/s41467-023-43324-w, published online 18 November 2023

In this article, the author names Paolo Tarantino, Hersh Gupta, Melissa E. Hughes, Janet Files, Sarah Strauss, Gregory Kirkner, Anne-Marie Feeney, Yvonne Li, Ana C. Garrido-Castro, Romualdo Barroso-Sousa, Brittany L. Bychkovsky, Simona DiLascio, Lynette Sholl, Laura MacConaill, Neal Lindeman, Bruce E. Johnson, Matthew Meyerson, Rinath Jeselsohn, Xintao Qiu, Rong Li, Henry Long, Eric P. Winer, Deborah Dillon, Giuseppe Curigliano, Andrew D. Cherniack, Sara M. Tolaney & Nancy U. Lin were incorrectly written as P. Tarantino, H. Gupta, M. E. Hughes, J. Files, S. Strauss, G. Kirkner, A.-M. Feeney, Y. Li, A. C. Garrido-Castro, R. Barroso-Sousa, B. L. Bychkovsky, S. DiLascio, L. Sholl, L. MacConaill, N. Lindeman, B. E. Johnson, M. Meyerson, R. Jeselsohn, X. Qiu, R. Li, H. Long, E. P. Winer, D. Dillon, G. Curigliano, A. D. Cherniack, S. M. Tolaney & N. U. Lin. The original article has been corrected.

