# Supplementary material for: Comprehensive genomic characterization of HER2-low and HER2-0 breast cancer
Source: Nat Commun. 2023 Nov 18;14:7496. doi: 10.1038/s41467-023-43324-w (PMC10657399; doi:10.1038/s41467-023-43324-w)
Supplement: Supplementary file 1 — Supplementary Information [file 41467_2023_43324_MOESM1_ESM.pdf]

SUPPLEMENTARY INFORMATION:

Comprehensive genomic characterization of HER2-low and HER2-0 breast cancer

P. Tarantino, H. Gupta, M.E.Hughes[1], J. Files, S.Strauss, G. Kirkner, A.-M. Feeney, Y. Li, A.C.Garrido-Castro, R. Barroso-Sousa, B.L. Bychkovsky, S. DiLascio, L. Sholl, L. MacConaill, N. Lindeman, B.E. Johnson, M. Meyerson, R. Jeselsohn, X. Qiu, R. Li, H. Long, E.P. Winer, D. Dillon, G. Curigliano, A.D. Cherniack, S.M. Tolaney, and N.U. Lin

This supplement contains the following information:

- **Supplementary Fig. 1.** REMARK diagram.
- **Supplementary Fig. 2.** Frequency of most common mutations (A) and copy number variations (B) among patients with metastatic disease at time of sample collection (n=801), divided by HER2-status and colored by ER status.
- **Supplementary Fig. 3.** Enrichment analysis of mutations (A) and copy number variation (CNV, B) among patients with metastatic disease at time of tissue collection (N=801).
- **Supplementary Fig. 4.** Comparison of ERBB2 copy counts between HER2-low and HER2-0 tumors among patients with metastatic disease at time of tissue collection (N=801).
- **Supplementary Fig. 5.** Frequency of most common mutations (A) and copy number variations (B) among patients with that were either IHC 0 or 2+ at time of sample collection (N = 759), divided by IHC (HER2) status and colored by ER status
- **Supplementary Fig. 6.** Enrichment analysis of mutations (A) and copy number variation (CNV, B) among patients with either IHC 0 or 2+ at time at time of tissue collection (N = 750).
- **Supplementary Fig. 7.** A plot of total TMB versus TMB that only resulted in an oncogenic mutation, colored by hypermutant status.
- **Supplementary Table 1**. Extended patient and tumor characteristics among patients with metastatic breast cancer included in the study.
- **Supplementary Table 2.** List of tumor suppressor genes that are further used to classify oncogenic mutations.
- **Supplementary Data File (attached Excel file).** Results of enrichment logistic regression. Included q-values were calculated separately for mutation and copy number events.

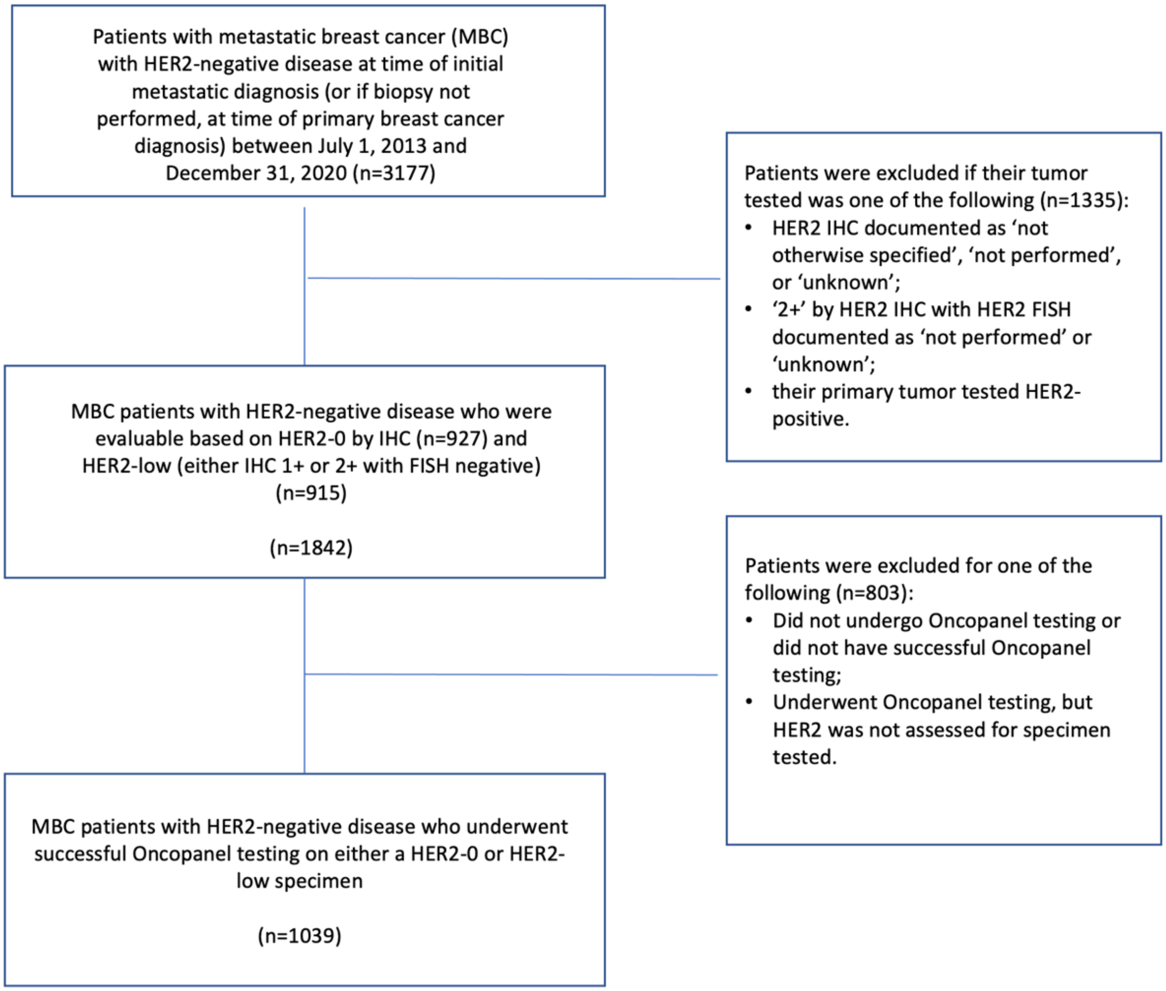

Supplementary Fig. 1. REMARK diagram

HER2, human epidermal growth factor receptor 2; **IHC**, immunohistochemistry

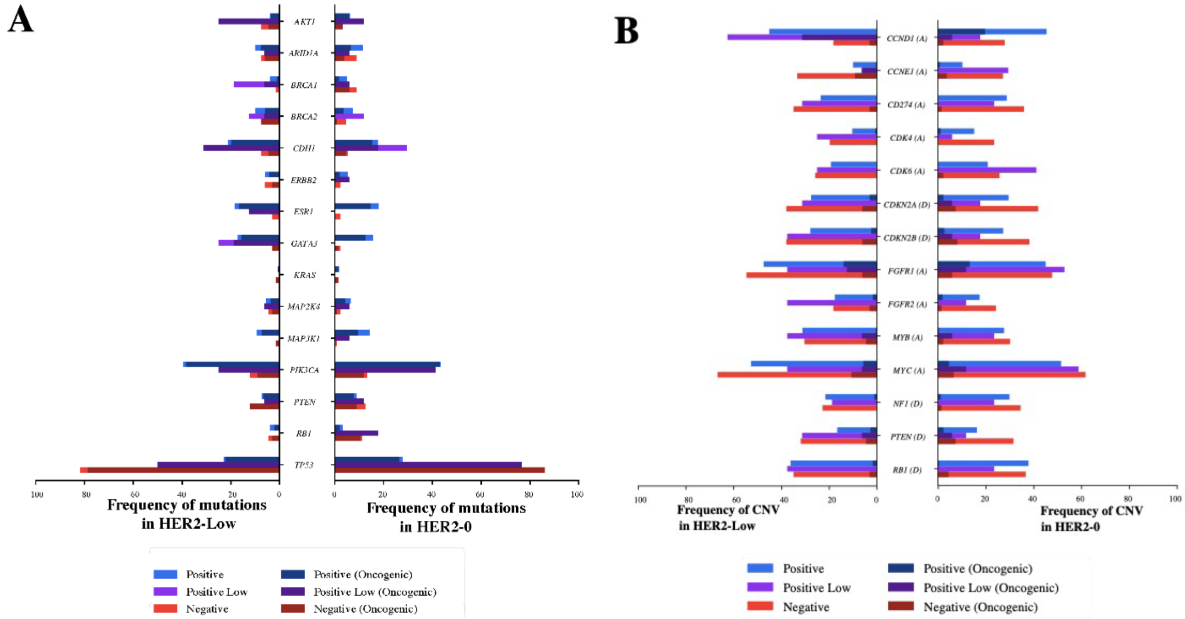

Supplementary Fig. 2. Frequency of most common mutations (A) and copy number variations (B) among patients with metastatic disease at time of sample collection (N = 801), divided by HER2-status and colored by ER status.

Shading represents the percentage of oncogenic events. In the right panel, an annotation of “(A)” beside a gene represents high amplification and “(D)” represents a deep or 2 copy deletion.

HER2, human epidermal growth factor receptor 2; **ER**, estrogen receptor

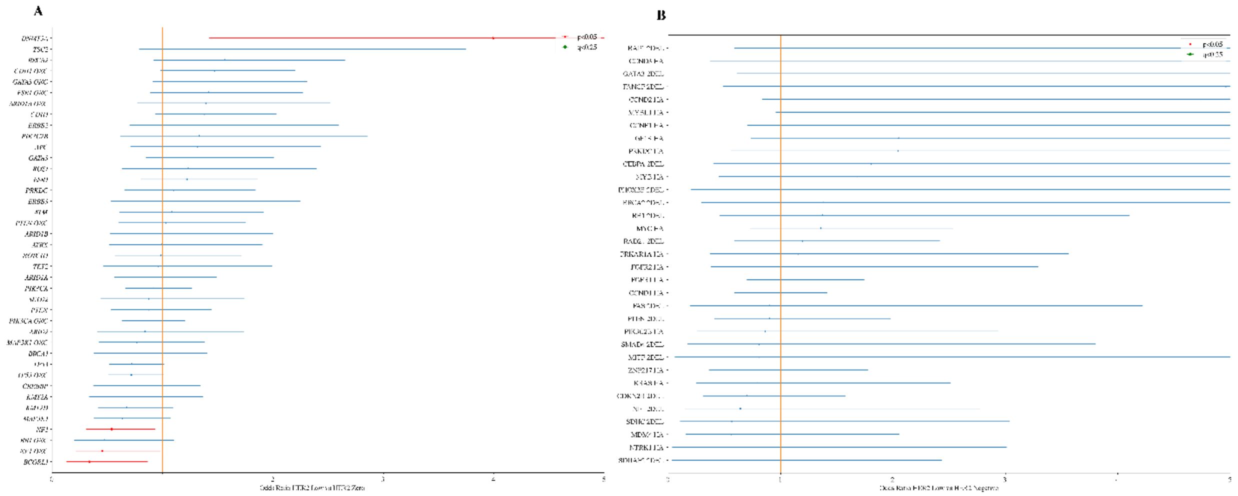

Supplementary Fig. 3. Enrichment analysis of mutations (A) and copy number variation (CNV, B) among patients with metastatic disease at time of tissue collection (N = 801).

Modeling was done using multivariate logistic regression accounting for ER status and background rate of either mutation or copy-number events, using the statsmodel package in Python. ER-low cases were included in the ER-positive group. Multiple hypothesis correction was done using FDR. Only models that reached a significant value under multiple hypothesis correction for rejecting the log-likelihood null were included, as well as those that converged after 500 iterations. Only mutations that appeared in over 4% of either all HER2-0 or HER2-low samples were included in this visualization. On the left, lines labeled "_ONC" represent only oncogenic mutations, while the CNVs were done on 2DELs or high amplifications for tumor suppressor genes and oncogenes, respectively (labeled on the figure). Error bars are reported as the 95% confidence interval. P-values are determined as the likelihood of the model's calculated coefficients under the assumption that the true coefficients are 0 and are reported as two-tailed. Multiple hypothesis correction was done using BH-FDR. Exact p-values are reported in the source data of this figure.

CNV, copy number variations; **ER,** estrogen receptor; **HER2,** human epidermal growth factor receptor 2

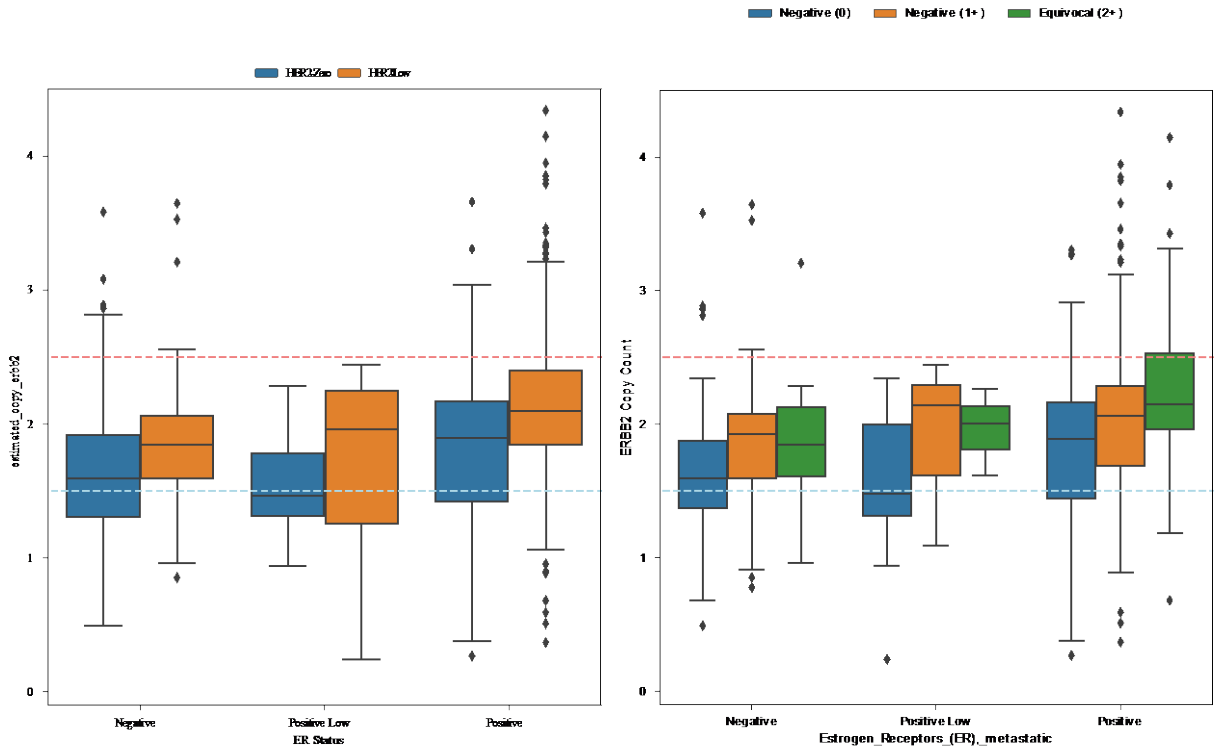

Supplementary Fig. 4. Comparison of ERBB2 copy counts between HER2-low and HER2-0 tumors among patients with metastatic disease at time of tissue collection (N = 575).

The left panel depicts the estimated copy count of ERBB2 by ER status and HER2 status. The blue line depicts the point at which HER2 would be called a 1-copy loss and the red line depicts the point at which it would be called a gain. On the right, a similar plot is shown, except colored by IHC staining for HER2. Box plots are constructed with the central line as the median, the outer lines of the box as the lower and upper quartile, and whiskers are equal to 1.5x the closest quartile.

HER2, human epidermal growth factor receptor 2; IHC, immunohistochemistry

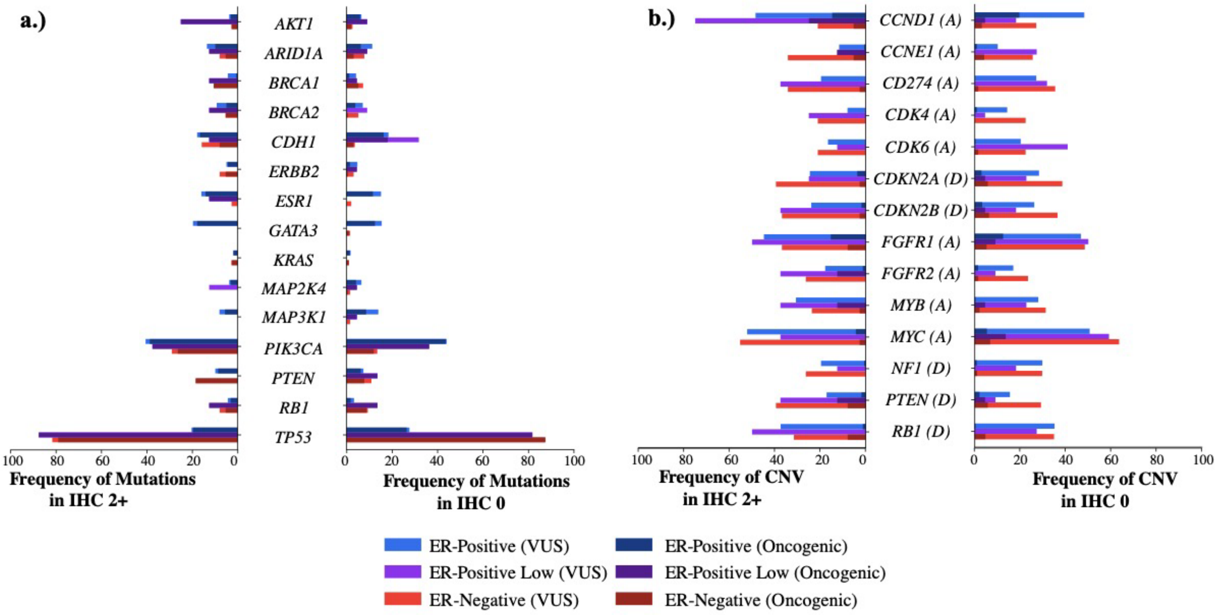

Supplementary Fig. 5. Frequency of most common mutations (a.) and copy number variations (b.) among patients with that were either IHC 0 or 2+ at time of sample collection (N = 759), divided by IHC (HER2) status and colored by ER status.

Shading represents the percentage of oncogenic events. In the right panel, an annotation of "(A)" beside a gene represents high amplification and "(D)" represents a deep or 2 copy deletion.

HER2, human epidermal growth factor receptor 2; **ER**, estrogen receptor

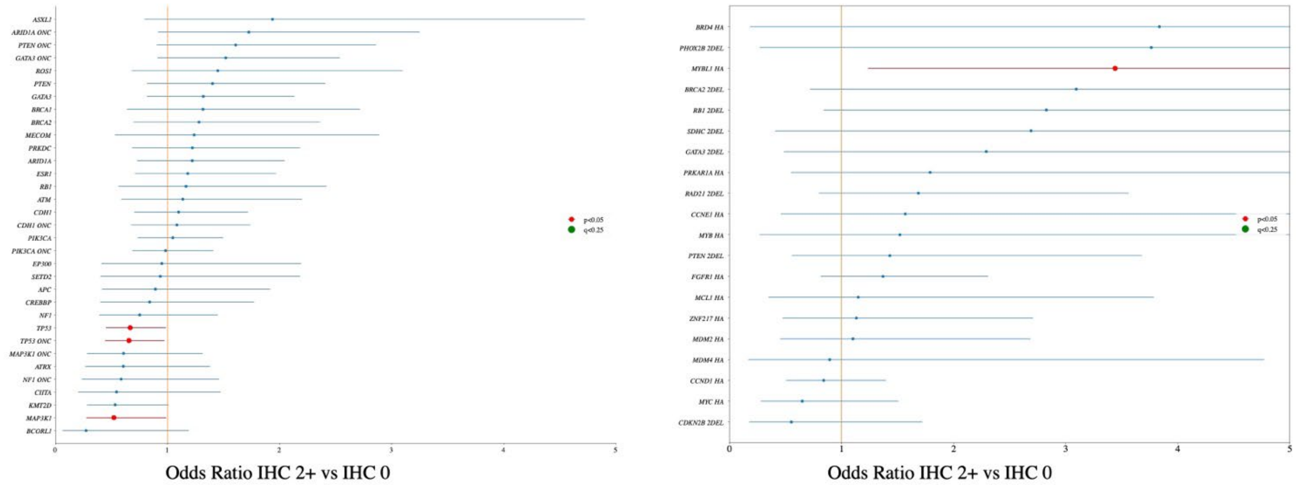

Supplementary Fig. 6. Enrichment analysis of mutations (left) and copy number variation (CNV, right) among patients with either IHC 0 or 2+ at time at time of tissue collection
(N = 750).

Modeling was done using multivariate logistic regression accounting for ER status and background rate of either mutation or copy-number events, using the statsmodel package in Python. ER-low cases were included in the ER-positive group. Multiple hypothesis correction was done using FDR. Only models that reached a significant value under multiple hypothesis correction for rejecting the log-likelihood null were included, as well as those that converged after 500 iterations. Only mutations that appeared in over 4% of either all HER2-0 or HER2-low samples were included in this visualization. On the left, lines labeled "_ONC" represent only oncogenic mutations, while the CNVs were done on 2DELs or high amplifications for tumor suppressor genes and oncogenes, respectively (labeled on the figure). Error bars are reported as the 95% confidence interval. P-values are determined as the likelihood of the model's calculated coefficients under the assumption that the true coefficients are 0 and are reported as two-tailed. Multiple hypothesis correction was done using BH-FDR. Exact p-values are reported in the source data of this figure.

CNV, copy number variations; **ER,** estrogen receptor; **HER2,** human epidermal growth factor receptor 2

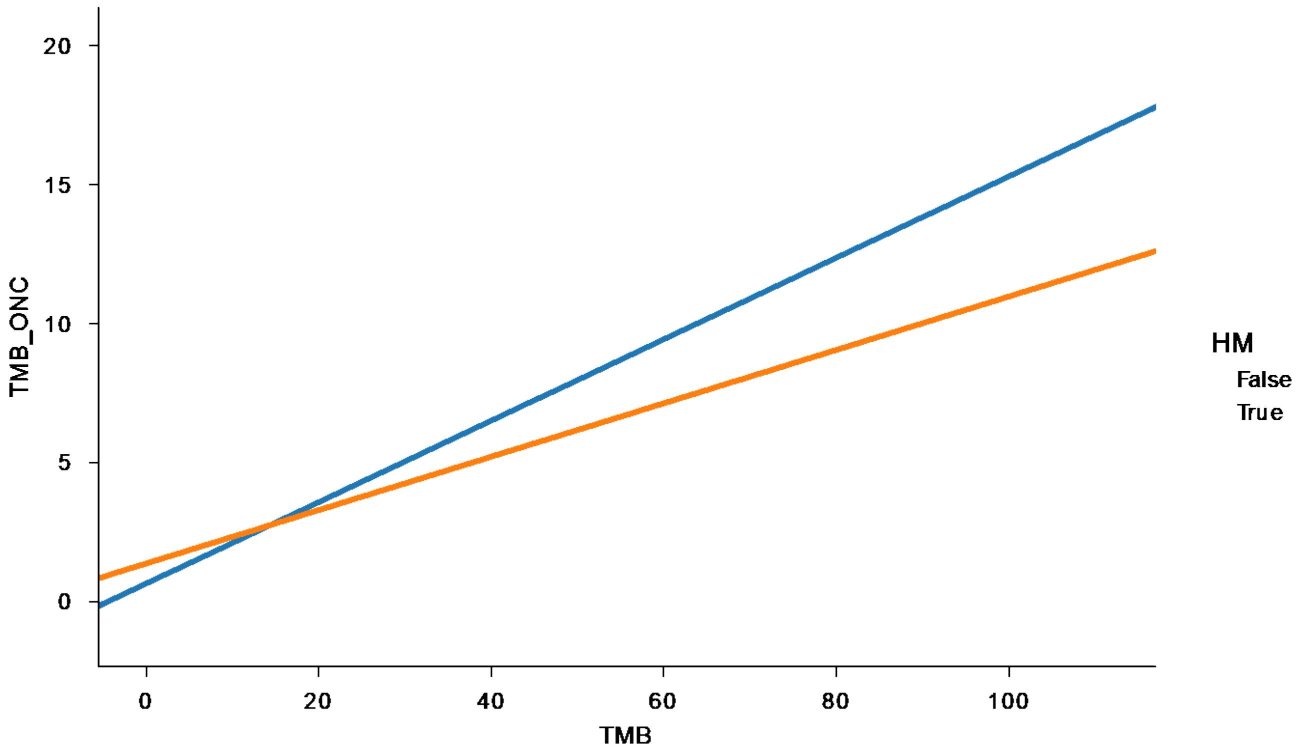

Supplementary Fig. 7. A plot of total TMB versus TMB that only resulted in an oncogenic mutation, colored by hypermutant status.

The estimated linear model depicts that, at lower number of total mutations, a higher proportion of the mutations are expected to be oncogenic. In particular, the slope of the hypermutant-estimated linear model diverges from the non-hypermutant, suggesting that this term should be included in the regression models.

TMB, tumor mutational burden

	Total Population (n=1039)	Patients with HER2-Low Tumors Tested (n=487)	Patients with HER2-0 Tumors Tested (n=552)	P-Value
**CNS Brain diagnosis,** *n (%)*	267 (25.7)	111 (22.8)	156 (28.3)	0.044
**Brain mets at time of initial met diagnosis,** *n (%)*	75 (7.2)	25 (5.1)	50 (9.1)	0.015
**Lung mets at time of initial met diagnosis,** *n (%)*	216 (20.8)	94 (19.3)	122 (22.1)	0.267
**Liver mets at time of initial met diagnosis,** *n (%)*	286 (27.5)	118 (24.2)	168 (30.4)	0.025
**Bone mets at time of initial met diagnosis,** *n (%)*	593 (57.1)	275 (56.5)	318 (57.6)	0.711
**Other mets at time of initial met diagnosis,** *n (%)*	635 (61.1)	301 (61.8)	334 (60.5)	0.668
**# Sites diagnosed at time of metastatic diagnosis,** *n (%)*	-	-	-	0.394
1	395 (38.0)	195 (40.0)	200 (36.2)	-
2	351 (33.8)	166 (34.1)	185 (33.5)	-
3	191 (18.4)	84 (17.3)	107 (19.4)	-
4+	102 (9.8)	42 (8.6)	60 (10.9)	-
**Received prior neo/adjuvant chemotherapy,** *n (%)**	617 (77.7)	271 (74.7)	346 (80.3)	0.06
**Received prior neo/adjuvant endocrine treatment,** *n (%)**	512 (64.5)	258 (71.1)	254 (58.9)	4E-04
**Received prior neo/adjuvant anti-HER2 treatment,** *n (%)**	18 (2.3)	9 (2.5)	9 (2.1)	0.71
**Metastatic Regimen Count at time of OncoPanel testing,** *median (min, max)***	0 (0, 10)	0 (0, 10)	0 (0, 10)	0.009
**Prior Treatments Received for metastatic disease at time of OncoPanel testing,** *n (%)*	-	-	-	-
Endocrine Therapy	513 (49.4)	259 (53.2)	254 (46.0)	0.02
Alpelisib	3 (0.3)	1 (0.2)	2 (0.4)	0.63
Everolimus	35 (3.4)	22 (4.5)	13 (2.4)	0.05
Immunotherapy	21 (2.0)	11 (2.3)	10 (1.8)	0.60
CDK4/6	137 (13.2)	70 (14.4)	67 (12.1)	0.28
Chemotherapy	604 (58.1)	273 (56.1)	331 (60.0)	0.20
ADCl	7 (0.7)	3 (0.6)	4 (0.7)	0.83

*Excludes those with Stage IV de-novo
**Excludes those with Procedure Dates Before Metastatic Diagnosis

HER2, human epidermal growth factor receptor 2; **CDK**, cyclin dependent kinase; **ADC**, antibody-drug conjugate

Supplementary Table 1. Extended patient and tumor characteristics among patients with metastatic breast cancer included in the study.

ACTG1	CDKN1B	ERCC6	INHA	NF2	RAD51B	SOCS3
AJUBA	CDKN1C	ERF	INHBA	NFKBIA	RAD51C	SOX17
ALOX12B	CDKN2A	ERRFI1	INPP4B	NFKBIE	RAD51D	SOX9
AMER1	CDKN2B	ESCO2	INPPL1	NKX3-1	RASA1	SP140
ANKRD11	CDKN2C	ETAA1	IRF1	NOTCH1	RB1	SPEN
APC	CEBPA	ETV6	IRF8	NOTCH2	RBL2	SPOP
APLNR	CHEK1	EXT1	JAK1	NOTCH3	RBM10	SPRED1
ARHGAP35	CHEK2	EXT2	JARID2	NOTCH4	RBM15	SPRTN
ARID1A	CIC	EZH2	KAT6B	NPM1	RECQL	STAG1
ARID1B	CIITA	FAM175A	KDM5C	NPRL2	RECQL4	STAG2
ARID2	CMTR2	FAM46C	KDM6A	NPRL3	RELA	STK11
ARID3A	COL7A1	FAM58A	KDM6B	NSD1	REST	SUFU
ARID4A	CRBN	FANCA	KEAP1	NTHL1	RHOH	SUZ12
ARID4B	CREBBP	FANCB	KLF2	P2RY8	RNF43	TBL1XR1
ARID5B	CRTC2	FANCC	KLF4	PALB2	ROBO1	TBX3
ASXL1	CTCF	FANCD2	KMT2A	PARK2	RTEL1	TCF3
ASXL2	CTNNA1	FANCE	KMT2B	PARP1	RUNX1	TCF7L2
ATM	CTR9	FANCF	KMT2C	PAX5	RYBP	TET1
ATP6V1B2	CUL3	FANCG	KMT2D	PBRM1	SAMHD1	TET2
ATR	CUX1	FANCI	LATS1	PDS5B	SBDS	TET3
ATRX	CYLD	FANCL	LATS2	PHF6	SDHA	TGFBR1
ATXN2	DAXX	FANCM	LRP5	PHOX2B	SDHAF2	TGFBR2
AXIN1	DDB2	FAS	LTB	PIGA	SDHB	TMEM127
AXIN2	DDX3X	FAT1	LZTR1	PIK3R1	SDHC	TNFAIP3
B2M	DDX41	FBXO11	MAD2L2	PIK3R2	SDHD	TNFRSF14
BACH2	DEPDC5	FBXW7	MAP2K4	PIK3R3	SERPINB3	TOP1
BAP1	DICER1	FH	MAP3K1	PMAIP1	SESN1	TP53
BARD1	DIS3	FLCN	MAX	PML	SESN2	TP53BP1
BBC3	DIS3L2	FOXA1	MBD4	PMS1	SESN3	TP63
BCL10	DKC1	FOXF1	MBD6	PMS2	SETD2	TRAF3
BCL11B	DNMT3A	FOXL2	MED12	POLB	SETDB1	TRAF5
BCL2L11	DNMT3B	FOXO1	MEN1	POLD1	SETDB2	TRIP13
BCOR	DTX1	FOXP1	MGA	POLE	SFRP1	TSC1
BCORL1	DUSP22	FUBP1	MITF	POLH	SFRP2	TSC2
BLM	DUSP4	GATA3	MLH1	POLQ	SH2B3	VHL
BMPR1A	ECT2L	GATA4	MLH3	POT1	SH2D1A	WIF1
BRCA1	EED	GATA6	MLL2	PPP2R1A	SHQ1	WRN
BRCA2	EGR1	GPC3	MOB3B	PPP2R2A	SLC34A2	WT1
BRCC3	ELF3	GPS2	MRE11A	PPP6C	SLFN11	XPA
BRIP1	ELMSAN1	GRIN2A	MSH2	PRDM1	SLX4	XPC
BTG1	EP300	HDAC4	MSH3	PTCH1	SMAD2	XRCC1
CASP8	EP400	HIST1H1B	MSH6	PTEN	SMAD3	XRCC2
CBFA2T3	EPCAM	HIST1H1D	MST1	PTPN1	SMAD4	XRCC3
CBFB	EPHA3	HLA-A	MTAP	PTPN2	SMARCA2	XRCC4
CBL	EPHA7	HLA-B	MUTYH	PTPRD	SMARCA4	XRCC5
CD58	EPHB1	HLA-C	NBN	PTPRS	SMARCB1	XRCC6
CDC73	ERCC1	HNF1A	NCOR1	PTPRT	SMARCE1	YWHAE
CDH1	ERCC2	HOXB13	NEIL1	QKI	SMC1A	ZBTB20
CDH4	ERCC3	ID3	NEIL2	RAD21	SMC3	ZFHX3
CDK12	ERCC4	IFNGR1	NEIL3	RAD50	SMG1	ZFP36L1
CDKN1A	ERCC5	IKZF3	NF1	RAD51	SOCS1	ZNRF3

Supplementary Table 2. List of tumor suppressor genes that are further used to classify oncogenic mutations.

Thesis list of genes was curated by a board-certified geneticist and a loss of function mutation in these genes was determined to be oncogenic.
