# Supplementary material for: Comprehensive genomic characterization of HER2-low and HER2-0 breast cancer
Source: Nat Commun. 2023 Nov 18;14:7496. doi: 10.1038/s41467-023-43324-w (PMC10657399; doi:10.1038/s41467-023-43324-w)
Supplement: Supplementary file 3 — Description of Additional Supplementary Files [file 41467_2023_43324_MOESM3_ESM.pdf]

Description of Additional Supplementary Files

File Name: Supplementary Data 1
Description: Results of enrichment logistic regression. Included q-values were calculated separately for mutation and copy number events.
