# Supplementary material for: Comprehensive genomic characterization of HER2-low and HER2-0 breast cancer
Source: Nat Commun. 2023 Nov 18;14:7496. doi: 10.1038/s41467-023-43324-w (PMC10657399; doi:10.1038/s41467-023-43324-w)
Supplement: Supplementary file 5 — Reporting Summary [file 41467_2023_43324_MOESM5_ESM.pdf]

nature portfolio

Corresponding author(s): Paolo Tarantino

Last updated by author(s): Oct 19, 2023

Reporting Summary

Nature Portfolio wishes to improve the reproducibility of the work that we publish. This form provides structure for consistency and transparency in reporting. For further information on Nature Portfolio policies, see our Editorial Policies and the Editorial Policy Checklist.

Statistics

For all statistical analyses, confirm that the following items are present in the figure legend, table legend, main text, or Methods section.

n/a	Confirmed	
☐	☒	The exact sample size (*n*) for each experimental group/condition, given as a discrete number and unit of measurement
☒	☐	A statement on whether measurements were taken from distinct samples or whether the same sample was measured repeatedly
☐	☒	The statistical test(s) used AND whether they are one- or two-sided<br>*Only common tests should be described solely by name; describe more complex techniques in the Methods section.*
☐	☒	A description of all covariates tested
☐	☒	A description of any assumptions or corrections, such as tests of normality and adjustment for multiple comparisons
☐	☒	A full description of the statistical parameters including central tendency (e.g. means) or other basic estimates (e.g. regression coefficient) AND variation (e.g. standard deviation) or associated estimates of uncertainty (e.g. confidence intervals)
☐	☒	For null hypothesis testing, the test statistic (e.g. *F*, *t*, *r*) with confidence intervals, effect sizes, degrees of freedom and *P* value noted<br>*Give P values as exact values whenever suitable.*
☒	☐	For Bayesian analysis, information on the choice of priors and Markov chain Monte Carlo settings
☒	☐	For hierarchical and complex designs, identification of the appropriate level for tests and full reporting of outcomes
☒	☐	Estimates of effect sizes (e.g. Cohen's *d*, Pearson's *r*), indicating how they were calculated

Our web collection on statistics for biologists contains articles on many of the points above.

Software and code

Policy information about availability of computer code

Data collection	Data was collected using the existing OncoPanel bioinformatics pipeline that has been described in detail. More details can be found in the submitted manuscript and at: Sholl et al., JCI Insight 2016, Ramkisoon et al., Neuro Oncology 2017, Hanna et al., JC Insight 2017
Data analysis	Analysis was carried out using the R statistical software package and Python and associated packages. Python version 3.10 with statsmodel package 0.13.1 and CMH package 1.0.1 and R version 4.3.1 and coin package 1.4.3 were used. Analysis for Table 1 was carried out

For manuscripts utilizing custom algorithms or software that are central to the research but not yet described in published literature, software must be made available to editors and reviewers. We strongly encourage code deposition in a community repository (e.g. GitHub). See the Nature Portfolio guidelines for submitting code & software for further information.

Data

Policy information about availability of data

All manuscripts must include a data availability statement. This statement should provide the following information, where applicable:

- Accession codes, unique identifiers, or web links for publicly available datasets
- A description of any restrictions on data availability
- For clinical datasets or third party data, please ensure that the statement adheres to our policy

The genomic data generated in this study has been deposited in AACR Project GENIE cBioPortal under the GENIE cohort public project. The genomic data in GENIE is under restricted access that can be accessed when agreeing to the terms and conditions of GENIE use. Clinical molecular, histological, and staging datasets and internally filtered genomic datasets are available in the Source Data with matching GENIE identifiers. However, only GENIE data has exact mutational data such as

base change. Exact mutations for samples missing from GENIE may be acquired from the authors, as well as additional clinical information if the researcher has proper access as described in the study protocol and the patient has agreed to sharing data with external entities.

Research involving human participants, their data, or biological material

Policy information about studies with human participants or human data. See also policy information about sex, gender (identity/presentation), and sexual orientation and race, ethnicity and racism.

Reporting on sex and gender	Sex was collected for all patients and reported in Table 1. Gender was not included as not applying to this specific genomic analysis.
Reporting on race, ethnicity, or other socially relevant groupings	All the relevant characteristics of the study population were carefully described in Table 1.
Population characteristics	All the relevant characteristics of the study population were carefully described in Table 1.
Recruitment	Participants with metastatic breast cancer being treated at Dana-Farber Cancer Institute from July 1, 2013 to December 31, 2020 were approached to have genomic profiling testing performed using next-generation sequencing platform (Oncopanel). If they provided consent to DF/HCC IRB #11-104 and/or #17-000, we included them in the study if they met inclusion criteria as stated in the manuscript.
Ethics oversight	Dana-Farber/Harvard Cancer Center Institutional Review Board

Note that full information on the approval of the study protocol must also be provided in the manuscript.

Field-specific reporting

Please select the one below that is the best fit for your research. If you are not sure, read the appropriate sections before making your selection.

[x] Life sciences [ ] Behavioural & social sciences [ ] Ecological, evolutionary & environmental sciences

For a reference copy of the document with all sections, see nature.com/documents/nr-reporting-summary-flat.pdf

Life sciences study design

All studies must disclose on these points even when the disclosure is negative.

Sample size	Given the retrospective nature of our study, all patients with a sample meeting inclusion criteria in our database were included.
Data exclusions	No data that met inclusion criteria was excluded from the study.
Replication	Replication was carried out by redoing the analysis in 2 smaller subpopulations of our study cohort. The first was patients that had a sample sequenced after a diagnosis of metastatic disease. The second was to only look at samples that were classified as IHC 0/2+, excluding samples that were classified as IHC 1+. Replication was successful and confirmed that in a more homogeneous population, our findings still held.
Randomization	As this was a retrospective study, randomization is not relevant as the investigators were not actively altering a variable and could not select which group participants would be selected in.
Blinding	As this was a retrospective study, blinding is not relevant as the investigators were not actively altering a variable and would not be able to introduce bias through knowledge of participants groups.

Reporting for specific materials, systems and methods

We require information from authors about some types of materials, experimental systems and methods used in many studies. Here, indicate whether each material, system or method listed is relevant to your study. If you are not sure if a list item applies to your research, read the appropriate section before selecting a response.

Materials & experimental systems

n/a	Involved in the study	
[x]	[ ]	Antibodies
[x]	[ ]	Eukaryotic cell lines
[x]	[ ]	Palaeontology and archaeology
[x]	[ ]	Animals and other organisms
[x]	[ ]	Clinical data
[x]	[ ]	Dual use research of concern
[x]	[ ]	Plants

Methods

n/a	Involved in the study	
[x]	[ ]	ChIP-seq
[x]	[ ]	Flow cytometry
[x]	[ ]	MRI-based neuroimaging

Plants

Seed stocks

Report on the source of all seed stocks or other plant material used. If applicable, state the seed stock centre and catalogue number. If plant specimens were collected from the field, describe the collection location, date and sampling procedures.

Novel plant genotypes

Describe the methods by which all novel plant genotypes were produced. This includes those generated by transgenic approaches, gene editing, chemical/radiation-based mutagenesis and hybridization. For transgenic lines, describe the transformation method, the number of independent lines analyzed and the generation upon which experiments were performed. For gene-edited lines, describe the editor used, the endogenous sequence targeted for editing, the targeting guide RNA sequence (if applicable) and how the editor was applied.

Authentication

Describe any authentication procedures for each seed stock used or novel genotype generated. Describe any experiments used to assess the effect of a mutation and, where applicable, how potential secondary effects (e.g. second site T-DNA insertions, mosiacism, off-target gene editing) were examined.
